# Supraspinal Activation Induced by Visual Kinesthetic Illusion Modulates Spinal Excitability

**DOI:** 10.3390/healthcare12171696

**Published:** 2024-08-26

**Authors:** Takeru Okouchi, Ryo Hirabayashi, Saki Nakashima, Asuka Abe, Hirotake Yokota, Chie Sekine, Tomonobu Ishigaki, Hiroshi Akuzawa, Mutsuaki Edama

**Affiliations:** Institute for Human Movement and Medical Sciences, Niigata University of Health and Welfare, Niigata 950-3198, Japan; hpm23013@nuhw.ac.jp (T.O.); rpa19086@nuhw.ac.jp (S.N.); rpa19004@nuhw.ac.jp (A.A.); yokota@nuhw.ac.jp (H.Y.); sekine@nuhw.ac.jp (C.S.); tomonobu-ishigaki@nuhw.ac.jp (T.I.); hiroshi-akuzawa@nuhw.ac.jp (H.A.); edama@nuhw.ac.jp (M.E.)

**Keywords:** F-wave, H-reflex, electrical stimulation, reciprocal inhibition, ankle joint

## Abstract

Repetitive passive movement (RPM) enhances reciprocal inhibition. RPM is more effective when performed rapidly and at wide joint angles. However, patients with limited joint range of motion may not receive the most effective RPM. Therefore, having an alternative method for performing RPM in patients who cannot perform actual exercise due to limited joint motion is necessary. This study investigated the effects of RPM on spinal excitability using a visual kinesthetic illusion. Participants included 17 healthy adults (7 women). Measurements were taken before, during, and immediately after the intervention. We established two intervention conditions: the control condition, in which participants focused their attention forward, and the illusion condition, in which participants watched a video about RPM. F-waves from the tibialis anterior and soleus muscles were measured, and F-wave persistence and F/M amplitude ratios were analyzed. Under the illusion condition, compared with the preintervention condition, the F/M amplitude ratio of the tibialis anterior increased by approximately 44% during the intervention (*p* < 0.05), whereas the F-wave persistence of the soleus decreased by approximately 23% from the immediate start of the intervention (*p* < 0.05). This study suggests that a visual kinesthetic illusion can increase the spinal excitability of the tibialis anterior, whereas reciprocal inhibition can decrease the spinal excitability of the soleus.

## 1. Introduction

Passive movement is widely used in rehabilitation. This movement can activate various areas of the brain, including the primary motor cortex, primary somatosensory cortex, supplementary motor cortex, posterior parietal cortex, and secondary somatosensory cortex [1,2,3,4,5]. Previous studies on repetitive passive movement (RPM) have shown a decrease in motor evoked potentials (MEPs), which indicates reduced excitability of the corticospinal tract [6,7,8,9]. Additionally, a study reported a decrease in MEPs with increasing RPM speed, although the F-wave, a measure of spinal excitability, remained unchanged [8]. This could be attributed to the postexercise cortical depression resulting from RPM. However, notably, during RPM, primary motor cortex excitability reportedly increases when participants focus on the moving object [9]. Our research group has previously focused on spinal inhibitory function, revealing an enhancement in spinal reciprocal inhibition after RPM [10,11]. These findings suggest that RPM is an effective intervention for patients with upper motor neuron disorders, such as stroke and spinal cord injury, in which spinal reciprocal inhibition is impaired. Furthermore, we revealed that the most effective kinematic parameters of RPM include a speed of 160°/s and a range of motion of 40° [10,11]. However, limitations in range of motion and heightened stretch reflexes caused by upper motor neuron dysfunction and impaired inhibitory function may hinder the effective use of these kinematic parameters, making it challenging to perform RPM optimally.

Therefore, we focused on utilizing a visual kinesthetic illusion. This phenomenon is defined as a psychological experience in which a person at rest feels the urge to move a body part while observing a video of that body part in motion [12]. A functional magnetic resonance imaging (fMRI) study investigating brain activity during a visual kinesthetic illusion of the wrist joint revealed activation of the premotor cortex, supplementary motor area, inferior parietal lobule, insular cortex, putamen, and other motion-related regions [12]. Additionally, a study measuring MEPs during a visual kinesthetic illusion of the ankle joint reported an increase in MEP amplitude during the intervention [13]. In a recent clinical study, visual kinesthetic illusion regarding ankle dorsiflexion was shown to increase muscle activation and improve standing motion in patients after stroke [14].

Similarly, motor imagery (MI) did not involve actual movement. MI can be defined as a dynamic state in which an individual mentally simulates a given action [15]. A previous fMRI study reported activity in the primary motor cortex, premotor cortex, and inferior parietal lobule during MI [16]. Additionally, Bunno et al. reported increased spinal cord excitability during MI about voluntary contractions [17]. A previous study of post-stroke participants reported that those who engaged in MI and daily rehabilitation improved their Fugl–Meyer assessment scores and balance and gait function [18]. However, despite numerous studies on the effects of a visual kinesthetic illusion and MI on brain and spinal cord functions, no study has investigated spinal cord excitability during visual kinesthetic illusory interventions. The effect of MI is also affected by the participant’s imaging ability [19,20]. Alternatively, the visual kinesthetic illusion modulates neural activity without any real joint movement by focusing on a video on a monitor [13,21]. While looking at the presented video, if participants observe an action by others, it is called action observation, something shown to increase corticospinal tract excitability similar to the visual kinesthetic illusion [22,23]. However, corticospinal tract excitability increases more effectively with the visual kinesthetic illusion than with action observation [21]. Therefore, a visual kinesthetic illusion is better than any other intervention to modulate neural activity without any real joint movement independent of the participant’s ability. Moreover, as visual kinesthetic illusions involve interventions using two-dimensional images, they can show high experimental reproducibility in experiments and be easily applicable in clinical settings. In addition, these interventions can be extended to others involving three-dimensional images, such as virtual reality.

Based on the above evidence, visual kinesthetic illusion interventions regarding RPM may enhance spinal function in patients who have difficulty performing RPM. Therefore, this study aimed to examine the effects of RPM without actual movement on spinal excitability using a visual kinesthetic illusion. We hypothesized that visual kinesthetic illusion involving the RPM increases spinal excitability in the tibial anterior (TA) muscle and soleus (Sol) muscle by increasing the number of descending pathways to anterior horn cells through the enhancement of primary motor cortex excitability.

## 2. Materials and Methods

### 2.1. Participants

In total, 17 healthy adults (10 men and 7 women; height, 164.8 ± 9.2 cm; weight, 55.1 ± 8.1 kg; age, 20.7 ± 1.2 years) were included in this study. We recruited the participants via e-mail sent to students at the Niigata University of Health and Welfare. The participants who had a history of lower extremity surgery, previous neuromuscular disease, psychiatric illness, or medication use were excluded from this study. The participants were fully informed about the research content and their rights, and written informed consent was obtained from them before study initiation. The study protocol was approved by the University’s Ethical Review Committee (18267-190918). The experiments were conducted in accordance with the ethical standards of the Niigata University of Health and Welfare and the 1964 Declaration of Helsinki and its later amendments.

### 2.2. Measurement Position

Measurements were conducted on the right lower limb, and the participants were asked to sit on a chair with 90° hip flexion, 60° knee flexion, and 20° ankle plantar flexion [10,11,24]. The lower limbs of the participants were fixed to the seat through the thighs and to the foot plate such that the limb position remained unchanged throughout the experiment (Takei scientific instruments, Niigata, Japan).

### 2.3. Electromyography (EMG)

Ag/AgCl electrodes (Blue Sensor, METS, Tokyo, Japan), at 20 mm between the electrodes, were used for EMG recording. The subjects were instructed to assume a prone position on the bed and perform ankle plantarflexion with the knee flexed to identify the soleus muscle. Next, the subjects were instructed to assume a supine position on the bed and perform ankle dorsiflexion to identify the tibialis anterior muscle. The electrodes were applied to the muscle belly of the right TA muscle and the muscle belly of the right Sol muscle, with the electrodes perpendicularly crossing the muscle fibers, according to SENIAM [25]. The skin underneath each electrode location was prepared and cleansed before sensor placement to reduce electrical impedance. A ground electrode was affixed between the stimulating electrode and the recording electrode of the TA muscle. Electromyographs were amplified 100-fold using an amplifier (FA-DL-720-140, 4Assist, Tokyo, Japan) and recorded on a personal computer at a sampling frequency of 10 kHz. The bandpass filter was set at 20–1000 Hz and raw waveforms were full-wave rectified. PowerLab 8/30 (AD Instruments, Colorado Spring, CO, USA) and LabChart 7 (AD Instruments) were used for analysis.

### 2.4. Experimental Procedure

**Figure 1:**
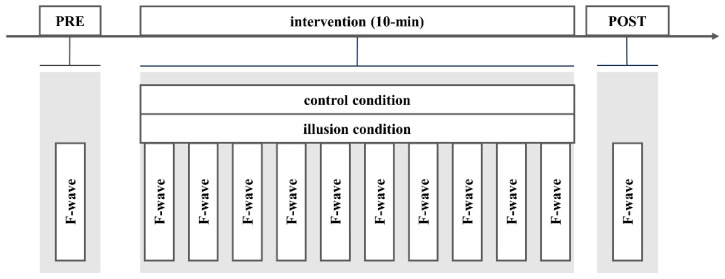
Experimental procedure.

We conducted a single-blind crossover study to investigate the effect of an intervention using a visual kinesthetic illusion involving RPM. All subjects participated at different times under both control and illusion conditions, but they were not informed about the conditions they were experiencing intervention. The experimental procedure is shown in Figure 1. To determine spinal cord excitability, F-waves were measured before (pre), during, and after (post) 10 intervention sets. A 50 s visual kinesthetic illusory intervention was followed by a 10 s rest period; each intervention set was performed for 1 min, resulting in a 10 min intervention. In previous studies on RPM, the intervention time was 10 min [6,8,10,11,24,26]. However, in the current study, considering the participants’ ability to concentrate during the illusion intervention, a break was provided during the intervention, resulting in 10 sets of interventions. There were two intervention conditions: no intervention (control condition) and illusion intervention (illusion condition). Our previous study found that focusing on the wall in front of them instead of the monitor during RPM did not influence spinal function [27]. Thus, in the control condition, the participants focused on a wall in front of them during the intervention. Conversely, in the illusion condition, the participants were exposed to a visual kinesthetic illusion during the intervention.

F-waves were measured at preintervention, during intervention, and postintervention.

Interventions (control and illusion conditions) were conducted randomly, and postintervention measurements were obtained immediately after the end of the intervention.

### 2.5. F-Wave Measurement

Stimulation electrodes were applied to the tibial and deep peroneal nerves. To measure evoked EMG signals, surface EMG electrodes were applied to the TA and Sol muscles at the abovementioned positions [25]. After applying the EMG electrodes, the participants performed active dorsiflexion and plantarflexion movements against manual resistance from the examiner. This process ensured the correct placement of the EMG electrodes. A previous study found that it was possible that H-reflex contaminated F-waves by the weak stimulation intensities [28,29,30], but higher stimulation intensities than Mmax induced the F-wave more reliably [30]. Therefore, we set the intensity to 120% of Mmax to ensure stimulation with an intensity that is definitely above Mmax [8,17]. Using a square wave, we set the stimulus intensity to a stimulus frequency of 0.5 Hz and a stimulus duration of 0.2 ms. During the intervention, 10 stimuli were delivered from each stimulating electrode per set, resulting in a total of 20 stimuli. Additionally, electrical stimulation was randomly applied 10 times each to the tibial and deep peroneal nerves.

### 2.6. Visual Kinesthetic Illusion

In the illusion condition, a visual kinesthetic illusion was displayed on a monitor. A previous study showed a correlation between the subjective degree of illusion (hereafter referred to as vividness) and the excitability of corticospinal tracts [13]. A previous study reported that visual kinesthetic illusion occurred in a video of the individual’s body but apparently did not appear in a video of another person’s body [31]. Therefore, we provided the visual kinesthetic illusion via a video of the participant’s foot on the intervention side, with the video playback monitor positioned above the participant’s own right foot. To create a sensation that the feet in the images belonged to their own bodies, the participants were asked to set the position of the monitor to the position of the participant’s own foot.

To exclude any effect of RPM on spinal excitability during video recording, an interval of ≥2 days was allowed between video recording and the beginning of the experiment. During the intervention, the participants were provided the following instruction: “Keep your eyes on the ankle joint movement in the video”.

### 2.7. Assessment of the Visual Kinesthetic Illusion

After implementing the illusory intervention, the vividness of the visual kinesthetic illusion was evaluated according to a previous study [12]. The statement “I had a feeling that my foot is actually moving during watching the movie” was scored on a seven-point Likert scale and used as the vividness of the illusion: −3, strongly disagree; −2, disagree; −1, somewhat disagree; 0, neither agree nor disagree; 1, somewhat agree; 2, agree; and 3, strongly agree.

### 2.8. RPM

RPM was performed using a passive movement control device (movement device; Takei scientific instruments, Niigata, Japan) to set the movement speed and joint angle. Based on previous studies, the motion speed was set at 160°/s, the motion range was set from 30° plantar flexion to 10° dorsiflexion of the ankle joint, and the starting limb position was set at 30° plantar flexion [10,11].

### 2.9. Data Analysis

Waveforms with a latency of <40 ms and peak-to-peak value of ≥35 µV were defined as F-waves; the peak-to-peak value of these waveforms was considered the F-wave amplitude. F-wave persistence was defined as the number of F-waves for which F-wave amplitude values could be measured [32]. The analysis items included the F/M amplitude ratio, F-wave persistence, and vividness of the visual kinesthetic illusion. The mean value of each analysis item was calculated for each set of interventions. The F-wave amplitude of waveforms with peak-to-peak values of <35 µV was set to 0 µV, and F-wave persistence was not recorded. Data during the intervention were calculated over five periods (Period 1–Period 5), with the data of one period being the mean of every two sets of data. The mean ± standard deviation values were calculated for the responses of all participants regarding the vividness of the visual kinesthetic illusion.

### 2.10. Statistical Processing

Statistical procedures included tests of normality and repeated measures of two-way analysis of variance (ANOVA) for condition and time factors. As post-tests, the comparisons of preintervention with during intervention and postintervention were performed using paired *t*-tests with Bonferroni correction, a statistical method that rigorously controls the false discovery rate. Additionally, since this study focused on comparisons with the PRE, Bonferroni-corrected paired *t*-tests were performed as post hoc analyses. The significance level was set at 5% in all comparisons.

## 3. Results

The background EMG for TA and Sol for each intervention condition and measurement time are shown in Table 1.

Regarding the F/M amplitude ratio of Sol, the results of repeated measures two-way ANOVA showed no main effect of condition (F[1, 16] = 0.687, *p* = 0.419, partial η^2^ = 0.041), no main effect of time (F[6, 96] = 0.409, *p* = 0.871, partial η^2^ = 0.025), and no interaction between the two factors (F[6, 96] = 0.940, *p* = 0.470, partial η^2^ = 0.055). Regarding the F/M amplitude ratio of TA, the results of repeated measures two-way ANOVA showed no main effect of condition (F[1, 16] = 0.182, *p* = 0.675, partial η^2^ = 0.011) and no main effect of time (F[6, 96] = 0.758, *p* = 0.605, partial η^2^ = 0.045) but showed an interaction between the two factors (F[6, 96] = 3.534, *p* = 0.003, partial η^2^ = 0.181).

Regarding F-wave persistence of Sol, the results of repeated measures two-way ANOVA showed no main effect of condition (F[1, 16] = 3.139, *p* = 0.096, partial η^2^ = 0.164) but showed a main effect of time (F[6, 96] = 2.792, *p* = 0.015, partial η^2^ = 0. 149). However, the results showed no interaction between the two factors (F[6, 96] = 3.736, *p* = 0.070, partial η^2^ = 0.112). Regarding F-wave persistence of TA, the results of repeated measures two-way ANOVA showed no main effect of condition (F[1, 16] = 0.057, *p* = 0.814, partial η^2^ = 0.004), no main effect of time (F[6, 96] = 0.263, *p* = 0.953, partial η^2^ = 0.016), and no interaction between the two factors (F[6, 96] = 1.724, *p* = 0.124, partial η^2^ = 0.097). The vividness of the visual kinesthetic illusion was 1.1 ± 0.3 on a seven-point Likert scale.

**Figure 2:**
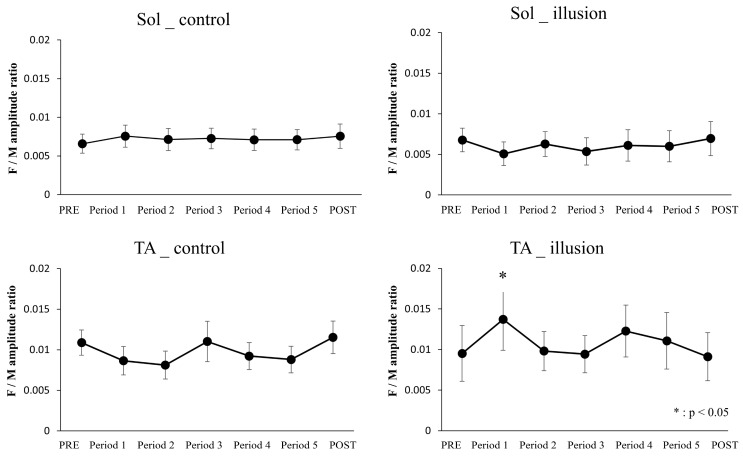
F/M amplitude ratio.

The values of the F/M amplitude ratio for each intervention condition and measurement time are presented in Table 2 and Figure 2.

The mean ± standard error of the F/M amplitude ratio of TA and Sol (Left, control condition; right, illusion condition). The vertical and horizontal axes indicate the F/M amplitude ratio and the results from pre- to postintervention, respectively. Data were analyzed by comparing the F/M amplitude ratio in PRE with the F/M amplitude ratio at each measurement time under the same intervention conditions. The F/M amplitude ratio of TA was significantly higher in Period 1 than in PRE (*p* < 0.05); * *p* < 0.05.

**Figure 3:**
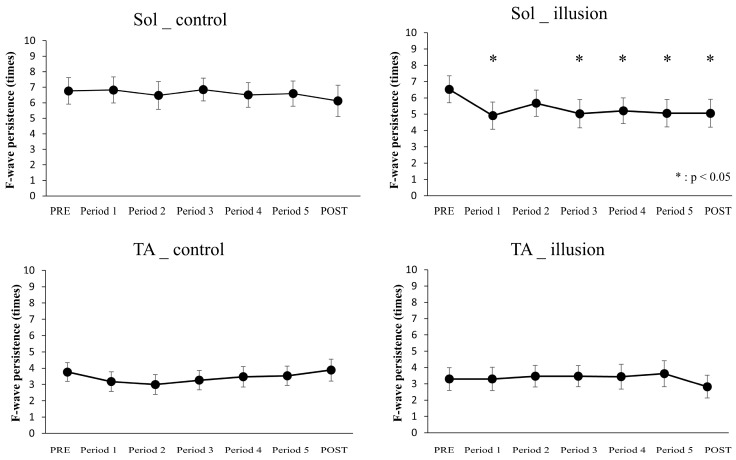
F-wave persistence. Mean ± standard error of the F-wave persistence of TA and Sol (**Left**, control condition; **right**, illusion condition). The vertical and horizontal axes indicate the F-wave persistence (times) and results from pre- to postintervention, respectively.

The F-wave persistence for each intervention condition and measurement time are presented in Table 3 and Figure 3.

Data were analyzed by comparing F-wave persistence in PRE with F-wave persistence at each measurement time under the same intervention conditions. The F-wave persistence in Sol was significantly lower in periods 1, 3, 4, 5, and POST than in PRE (*p* < 0.05); * *p* < 0.05.

## 4. Discussion

This study examined the effects of a visual kinesthetic illusion about RPM on spinal excitability. This study revealed two main findings. First, in the illusion condition, the F/M amplitude of TA significantly increased immediately after the start of the intervention. Second, the F-wave persistence of Sol significantly decreased during and after postintervention. The F/M amplitude and F-wave persistence indicate the excitability of spinal motoneurons [33,34,35,36]. Thus, the spinal excitability of TA significantly increased immediately after the start of the intervention, whereas that of Sol significantly decreased during the intervention. No previous studies had reported spinal cord excitability during a visual kinesthetic illusion, and a previous study examining spinal cord excitability before and after an illusory intervention reported no change in spinal cord excitability [37]. Thus, the current study provides new insights into spinal cord excitability during a visual kinesthetic illusory intervention.

As shown in the results, the F/M amplitude ratio of the TA showed no significant differences in the control condition; however, a significant difference was observed in Period 1 compared with PRE in the illusion condition. Therefore, the interaction in the F/M amplitude ratio of the TA results from an increase in the F/M amplitude ratio in the illusion condition. An explanation for these results is that the illusion of RPM may have simulated an RPM intervention with foot gazing. In this study, the visual kinesthetic illusory intervention involved a video about RPM of the ankle joint. Tsuiki et al. reported that RPM can increase corticospinal tract excitability by gazing at the intervention target [9]. Gazing has been reported to decrease SAI [38]. SAI represents inhibition of the primary motor cortex excitability due to sensory input [39] and involves GABAergic activity [40]. Supporting this evidence, short-interval intracortical inhibition [41,42], which is associated with GABAergic inhibitory mechanisms, reportedly decreases with gazing. On the basis of this evidence, it is possible that the visual kinesthetic illusion of RPM led to an increase in primary motor cortex excitability due to a decrease in cortical inhibitory circuits caused by the focus on the dorsal area of the foot displayed on the monitor. Similar to the current study, a previous study using a visual kinesthetic illusion about active plantar and dorsiflexion motion of the ankle joint reported significantly increased MEP amplitude of TA after the illusion [13]. On the other hand, the MEP amplitude of Sol does not change significantly after the illusion of ankle plantarflexion [13]. In this study, an increase in spinal excitability was observed only in the TA, suggesting that enhancement of the MEP amplitude may appear in the specific area that is focused on during the visual kinesthetic illusion. The subjective evaluation of the vividness of the illusion in the dorsal flexion and flexion directions was conducted using a visual analog scale; a moderate correlation was reported between the vividness of the visual kinesthetic illusion in the dorsiflexion direction and corticospinal tract excitability [13]. In addition, the vividness of the visual kinesthetic illusion in the current study is similar to that reported in a previous study [13]. Based on these findings, we suggest that a highly vivid illusion of ankle dorsal flexion resulted in increased attention on the dorsal foot, which in turn led to increased spinal excitability of the TA by decreasing the intracortical inhibitory circuit excitability. Individual differences in the effect of the visual kinesthetic illusion of RPM on spinal excitability may be attributed to variations in the vividness of the illusion, which could affect the degree of motor cortical excitability.

The present study results differed from our hypothesis regarding the significant decrease in the F-wave persistence of Sol immediately after the initiation of the intervention. According to this result, we suggest that the increase in the spinal excitability of TA observed during the visual kinesthetic illusion using RPM videos causes reciprocal inhibition on Sol. Previous studies on RPM reported enhancement of reciprocal inhibition 5 to 15 min after the intervention [10,11,24]. However, in a previous study examining the effects of transcranial direct current stimulation (tDCS) on spinal reciprocal inhibition, reciprocal inhibition was enhanced immediately after the start of tDCS [11,24]. Thus, in RPM, the muscle spindle firing signal from voluntary movement ascends to the brain, causing a delay until the occurrence of reciprocal inhibition enhancement. Conversely, this process does not occur when the brain is stimulated, and the enhancement of reciprocal inhibition can occur immediately. Thus, the present study suggests that spinal reciprocal inhibition is associated with increased spinal excitability of TA because the visual kinesthetic illusion may have decreased the spinal excitability of Sol over the following 10 min.

Our results suggest that a visual kinesthetic illusion can modulate spinal excitability without real joint movement and indicate the possibility of enhancing reciprocal inhibition. Future research should explore the optimal duration of interventions, identify parameters that sustain effects after treatment, and investigate whether or not motor function improves in patients with upper motor neuron disorders.

This study has several limitations. First, the sample size was determined on the basis of power analysis estimates. In addition, as only healthy young adults were recruited, recruitment bias was possible. To address these limitations, future research should include a large sample size, including older adults. Second, given the lack of a sham condition in the intervention condition, the effects of gazing at the monitor displaying the foot on spinal excitability remain unclear. In addition, subjects may have developed potential biases regarding the intervention condition. Therefore, future studies should consider including a sham condition. Third, whether the modulation of spinal function is attributable to gazing at the dorsal part of the foot remains unclear. Fourth, because the spinal excitability of TA and Sol were investigated concurrently and over different periods, fewer stimuli were used than in previous studies on F-wave measurements. Future research will involve a more detailed evaluation of the illusion and the presence or absence of enhanced reciprocal inhibition.

## 5. Conclusions

This study investigated the effect of a visual kinesthetic illusion regarding RPM for spinal excitability in the TA and Sol. The results showed that a visual kinesthetic illusion regarding RPM increased the spinal excitability of TA but decreased it for Sol. The visual kinesthetic illusion and focus on the monitor increased supraspinal excitability and modulated spinal function. In the future, research focusing on the effects of visual kinesthetic illusions on the inhibitory functions of the central nervous system may reveal the potential of visual motion illusions regarding RPM as a clinical intervention to improve the joint range of motion and enhance motor function in patients with limited physical activity.

## Figures and Tables

**Table 1:** Background EMG (µV).

		PRE	Period 1	Period 2	Period 3	Period 4	Period 5	POST
Sol	control	4.2 ± 0.5	4.5 ± 0.6	4.3 ± 0.5	4.1 ± 0.5	4.1 ± 0.5	4.9 ± 1.0	3.4 ± 0.5
	illusion	4.3 ± 0.6	5.0 ± 0.8	4.6 ± 0.6	4.7 ± 0.6	4.7 ± 0.6	7.4 ± 3.3	4.3 ± 0.6
TA	control	10.6 ± 1.7	10.5 ± 1.7	8.0 ± 1.6	10.5 ± 1.7	10.6 ± 1.7	10.7 ± 1.7	10.7 ± 1.7
	illusion	22.8 ± 9.8	14.0 ± 1.4	14.1 ± 1.4	13.7 ± 1.5	13.7 ± 1.5	13.7 ± 1.5	13.5 ± 1.7

Data are presented as the mean ± standard error. TA background EMG (µV) (EMG 30–50 ms before test stimulus).

**Table 2:** F/M amplitude ratio.

		PRE	Period 1	Period 2	Period 3	Period 4	Period 5	POST
Sol	control	6.6 ± 1.2	7.3 ± 1.4	7.1 ± 1.4	7.3 ±1.4	7.1 ± 1.4	7.1 ± 1.3	7.6 ± 1.6
	illusion	6.8 ± 1.5	5.0 ± 1.4	6.3 ± 1.5	5.3 ± 1.7	6.1 ± 1.9	6.0 ± 1.9	7.0 ± 2.1
TA	control	10.9 ± 1.6	8.9 ± 1.8	9.3 ± 1.8	11.0 ± 2.4	9.2 ± 1.7	9.2 ± 1.7	11.5 ± 2.0
	illusion	9.5 ± 3.4	13.7 ± 3.8 *	9.8 ± 2.4	9.4 ± 2.2	12.2 ± 3.2	11.1 ± 3.4	9.1 ± 3.0

Data are presented as the mean ± standard error. The table presents the result of each measurement time for each intervention condition. The value is the F-wave/Mmax. * *p* < 0.05 (Paired *t*-test with Bonferroni correction)

**Table 3:** Sol F-wave persistence.

		PRE	Period 1	Period 2	Period 3	Period 4	Period 5	POST
Sol	control	6.8 ± 0.8	6.8 ± 0.8	6.5 ± 0.9	6.9 ± 0.7	6.5 ± 0.8	6.6 ± 0.8	6.1 ± 1.0
	illusion	6.5 ± 0.8	4.9 ± 0.8 *	5.7 ± 0.8	5.0 ± 0.9 *	5.2 ± 0.8 *	5.1 ± 0.8 *	5.1 ± 0.9 *
TA	control	3.8 ± 0.6	3.2 ± 0.6	3.0 ± 0.6	3.4 ± 0.6	3.5 ± 0.6	2.1 ± 0.6	3.9 ± 0.7
	illusion	3.3 ± 0.7	3.3 ± 0.7	3.5 ± 0.7	3.5 ± 0.6	3.4 ± 0.8	3.6 ± 0.8	2.8 ± 0.7

Data are presented as the mean ± standard error. The table presents the result of each measurement time for each intervention condition. The value is for F-wave persistence. * *p* < 0.05 (Paired *t*-test with Bonferroni correction

## Data Availability

The raw data supporting the conclusions of this article will be made available by the authors upon request.

## References

[1] Weiller C., Jüptner M., Fellows S., Rijntjes M., Leonhardt G., Kiebel S., Müller S., Diener H.C., Thilmann A.F. (1996). Brain representation of active and passive movements. Neuroimage.

[2] Alary F., Doyon B., Loubinoux I., Carel C., Boulanouar K., Ranjeva J.P., Celsis P., Chollet F. (1998). Event-related potentials elicited by passive movements in humans: Characterization, source analysis, and comparison to fMRI. Neuroimage.

[3] Reddy H., Floyer A., Donaghy M., Matthews P.M. (2001). Altered cortical activation with finger movement after peripheral denervation: Comparison of active and passive tasks. Exp. Brain Res..

[4] Radovanovic S., Korotkov A., Ljubisavljevic M., Lyskov E., Thunberg J., Kataeva G., Danko S., Roudas M., Pakhomov S., Medvedev S. (2002). Comparison of brain activity during different types of proprioceptive inputs: A positron emission tomography study. Exp. Brain Res..

[5] Onishi H., Sugawara K., Yamashiro K., Sato D., Suzuki M., Kirimoto H., Tamaki H., Murakami H., Kameyama S. (2013). Neuromagnetic activation following active and passive finger movements. Brain Behav..

[6] Miyaguchi S., Onishi H., Kojima S., Sugawara K., Tsubaki A., Kirimoto H., Tamaki H., Yamamoto N. (2013). Corticomotor excitability induced by anodal transcranial direct current stimulation with and without non-exhaustive movement. Brain Res..

[7] Otsuka R., Sasaki R., Tsuiki S., Kojima S., Onishi H. (2017). Post-exercise cortical depression following repetitive passive finger movement. Neurosci. Lett..

[8] Sasaki R., Nakagawa M., Tsuiki S., Miyaguchi S., Kojima S., Saito K., Inukai Y., Masaki M., Otsuru N., Onishi H. (2017). Regulation of primary motor cortex excitability by repetitive passive finger movement frequency. Neuroscience.

[9] Tsuiki S., Sasaki R., Pham M.V., Miyaguchi S., Kojima S., Saito K., Inukai Y., Otsuru N., Onishi H. (2019). Repetitive passive movement modulates corticospinal excitability: Effect of movement and rest cycles and subject attention. Front. Behav. Neurosci..

[10] Hirabayashi R., Edama M., Kojima S., Miyaguchi S., Onishi H. (2019). Effects of repetitive passive movement on ankle joint on spinal reciprocal inhibition. Exp. Brain Res..

[11] Hirabayashi R., Edama M., Kojima S., Miyaguchi S., Onishi H. (2020). Enhancement of spinal reciprocal inhibition depends on the movement speed and range of repetitive passive movement. Eur. J. Neurosci..

[12] Kaneko F., Shindo K., Yoneta M., Okawada M., Akaboshi K., Liu M. (2019). A case series clinical trial of a novel approach using augmented reality that inspires self-body cognition in patients with stroke: Effects on motor function and resting-state brain functional connectivity. Front. Syst. Neurosci..

[13] Aoyama T., Kaneko F., Hayami T., Shibata E. (2012). The effects of kinesthetic illusory sensation induced by a visual stimulus on the corticomotor excitability of the leg muscles. Neurosci. Lett..

[14] Tanabe J., Amimoto K., Sakai K., Morishita M., Osaki S., Yoshihiro N., Kataoka T. (2023). Effects of visual-motor illusions with different visual stimuli on the sit-to-stand of people with hemiplegia following stroke: A randomized crossover controlled trial. Hum. Mov. Sci..

[15] Decety J. (1996). The neurophysiological basis of motor imagery. Behav. Brain Res..

[16] Mizuguchi N., Kanosue K. (2017). Changes in brain activity during action observation and motor imagery: Their relationship with motor learning. Prog. Brain Res..

[17] Bunno Y., Onigata C., Suzuki T. (2015). Excitability of spinal motor neurons during motor imagery of thenar muscle activity under maximal voluntary contractions of 50% and 100. J. Phys. Ther. Sci..

[18] Cho H.Y., Kim J.S., Lee G.C. (2013). Effects of motor imagery training on balance and gait abilities in post-stroke patients: A randomized controlled trial. Clin. Rehabil..

[19] Sharma N., Pomeroy V.M., Baron J.C. (2006). Motor imagery: A backdoor to the motor system after stroke?. Stroke.

[20] Almufareh M.F., Kausar S., Humayun M., Tehsin S. (2023). Leveraging motor imagery rehabilitation for individuals with disabilities: A review. Healthcare.

[21] Kaneko F., Yasojima T., Kizuka T. (2007). Kinesthetic illusory feeling induced by a finger movement movie effects on corticomotor excitability. Neuroscience.

[22] Nojima I., Koganemaru S., Kawamata T., Fukuyama H., Mima T. (2015). Action observation with kinesthetic illusion can produce human motor plasticity. Eur. J. Neurosci..

[23] Bisio A., Biggio M., Avanzino L., Ruggeri P., Bove M. (2019). Kinaesthetic illusion shapes the cortical plasticity evoked by action observation. J. Physiol..

[24] Hirabayashi R., Kojima S., Edama M., Onishi H. (2020). Activation of the supplementary motor areas enhances spinal reciprocal inhibition in healthy individuals. Brain Sci..

[25] Hermens H.J., Freriks B., Disselhorst-Klug C., Rau G. (2000). Development of recommendations for SEMG sensors and sensor placement procedures. J. Electromyogr. Kinesiol..

[26] Sasaki R., Tsuiki S., Miyaguchi S., Kojima S., Saito K., Inukai Y., Otsuru N., Onishi H. (2018). Repetitive passive finger movement modulates primary somatosensory cortex excitability. Front. Hum. Neurosci..

[27] Hirabayashi R., Edama M., Takeda M., Yamada Y., Yokota H., Sekine C., Onishi H. (2023). Participant attention on the intervention target during repetitive passive movement improved spinal reciprocal inhibition enhancement and joint movement function. Eur. J. Med. Res..

[28] Hagbarth K.E. (1962). Post-tetanic potentiation of myotatic reflexes in man. J. Neurol. Neurosurg. Psychiatry.

[29] Upton A.R., McComas A.J., Sica R.E. (1971). Potentiation of “late” responses evoked in muscles during effort. J. Neurol. Neurosurg. Psychiatry.

[30] Fisher M.A. (2007). F-waves—Physiology and clinical uses. Sci. World J..

[31] Kaneko F., Blanchard C., Lebar N., Nazarian B., Kavounoudias A., Romaiguère P. (2015). Brain regions associated to a kinesthetic illusion evoked by watching a video of one’s own moving hand. PLoS ONE.

[32] Weber F. (1998). The diagnostic sensitivity of different F wave parameters. J. Neurol. Neurosurg. Psychiatry.

[33] Fisher M.A. (1980). Relative changes with contraction in the central excitability state of the tibialis anterior and calf muscles. J. Neurol. Neurosurg. Psychiatry.

[34] Mercuri B., Wassermann E.M., Manganotti P., Ikoma K., Samii A., Hallett M. (1996). Cortical modulation of spinal excitability: An F-wave study. Electroencephalogr. Clin. Neurophysiol..

[35] Fisher M.A. (1998). The contemporary role of F-wave studies. F-wave studies: Clinical utility. Muscle Nerve..

[36] Rivner M.H. (2008). The use of F-waves as a probe for motor cortex excitability. Clin. Neurophysiol..

[37] Aoyama T., Kanazawa A., Kohno Y., Watanabe S., Tomita K., Kaneko F. (2021). Influence of visual stimulation-induced passive reproduction of motor images in the brain on motor paralysis after stroke. Front. Hum. Neurosci..

[38] Mirdamadi J.L., Suzuki L.Y., Meehan S.K. (2017). Attention modulates specific motor cortical circuits recruited by transcranial magnetic stimulation. Neuroscience.

[39] Turco C.V., El-Sayes J., Savoie M.J., Fassett H.J., Locke M.B., Nelson A.J. (2018). Short- and long-latency afferent inhibition; uses, mechanisms and influencing factors. Brain Stimul..

[40] Ziemann U., Reis J., Schwenkreis P., Rosanova M., Strafella A., Badawy R., Müller-Dahlhaus F. (2015). TMS and drugs revisited 2014. Clin. Neurophysiol..

[41] Ziemann U., Lönnecker S., Steinhoff B.J., Paulus W. (1996). Effects of antiepileptic drugs on motor cortex excitability in humans: A transcranial magnetic stimulation study. Ann. Neurol..

[42] Ziemann U., Lönnecker S., Steinhoff B.J., Paulus W. (1996). The effect of lorazepam on the motor cortical excitability in man. Exp. Brain Res..

